# Supplementary material for: Concerns related to returning home to a “difficult-to-return zone” after a long-term evacuation due to Fukushima Nuclear Power Plant Accident: A qualitative study
Source: PLoS One. 2022 Aug 29;17(8):e0273684. doi: 10.1371/journal.pone.0273684 (PMC9423635; doi:10.1371/journal.pone.0273684)
Supplement: S1 Table — (PDF) [file pone.0273684.s001.pdf]

Supplement S1 Table. The SCAT coding form

No	Speaker	Text	<1> Noteworthy words or phrases from the text	<2> paraphrazes of <1>	<3> concepts from out of the text that account for <2>	<4> themes, constructs in considerations of context	<5> questions & tasks
1	Mr.X (Feb 9 2022)	My family and I live in a city away from Iitate Village, so the foundation of living is no longer in the Village. Since 10 years have already passed since the disaster, I inevitably feel I have emigrated. Maintaining the community activities in the Nagadoro district, which used to be a peaceful farming community, is very difficult for residents.	Since 10 years have already passed since the disaster; Maintaining the community activities in the Nagadoro district, which used to be a peaceful farming community, is very difficult for residents.	change in living foundation due to long-term evacuation; recognition that migration is unavoidable; membership of the Village; difficulty in rebuilding and maintaining community activities in the Nagadoro district	changes in living base and base of operations due to changes and prolonged evacuation; membership as diverse residents; a sense of time 10 years after the nuclear accident	impossibility of rebuilding and maintaining the community; The passage of time since the FDNPP accident	
2	Mr.X (Feb 9 2022)	Then, it is hard to find people who would rebuild a house and live there.	it is hard to find people; ...rebuild a house and live there.	need to secure a house in Nagadoro district; the reality that the original house is not usable; absence of people who are willing to return whatever it takes	long-term prohibition of entry due to designation as a difficult-to-return zone; dilapidation or collapse of the original house; the necessity of building a new dwelling; cost burden; a choice not to return	long-term prohibition of entry due to its designation as a difficult-to-return zone; significant deterioration and collapse of their former dwellings; the cost of rebuilding new dwellings to live in after their return; rational decision of returning or not	
3	Mr.X (Feb 9 2022)	It is unlikely that anyone would be willing to come back here to live and farm.	It is unlikely that anyone would be willing to come back here; ...live and farm.	agriculture as an essential local industry; the impossibility of farming; absence of people to resume farming	Nagadoro district, which was centred on farming, a primary industry; agriculture with work and residence close to each other; damage to livelihood infrastructure; Villagers who do not intend to return	Nagadoro district as an agriculture-oriented area; prospects that residents hardly return	
4	Mr.X (Feb 9 2022)	Residents in our area were also concerned about the water supply, as they were using well water. Some households used to draw water from wells or rivers, but they were too afraid to drink it anymore after the nuclear accident.	using well water; they were too afraid to drink it anymore.	difficulty in securing water sources; health and safety concerns due to radioactive contamination of draw water	water as a necessity for daily life; environmental contamination due to lack of radiation decontamination in mountains and forests; risk of radioactive contamination of water in wells and river	wells and river water at risk of radiation contamination; difficulty of securing water sources; unsustainable livelihoods	
5	Mr.X (Dec 6 2021)	Suppose that, after setting up a reconstruction base in the Nagadoro district and proceeding with decontamination work by the government, the evacuation order is lifted, and the residents are ready to return to the district. But what if, in fact, not even 10% of them return? What if, in the worst case, only a handful of them returned to their homes? According to the government's explanation, it is assumed that 70% of residents will return.	But what if, in fact, not even 10% of them return?	decontamination associated with reconstruction projects; return after decontamination; government projections and policies regarding the return of residents;	decontamination as a part of the total recovery plan; few returnees; decreased motivation and interest in recovery among public	Nagadoro district as a model case or touchstone for promoting return; obstacle to decontamination and reconstruction efforts in other evacuation zones	
6	Mr.X (Dec 6 2021)	The cost for reconstruction in the Nagadoro district is already in the tens of billions of yen. If the residents do not return, the consequence will be a recognition among the public that there was no need to make an effort and support recovery since the residents did not want what the government was trying to do.	The cost for reconstruction in the Nagadoro district is already in the tens of billions of yen; there was no need to make an effort and support recovery since the residents did not want what the government was trying to do.	decontamination carried out at taxpayer expense; a number of returnees as a result of decontamination; feeling sorry for the relationship between cost and returning	reconstruction, decontamination, and return as a major national project; small number of returnees; project evaluation; enormous reconstruction budget	decontamination work as a government project funded by taxes; evaluation by the number of people who return	
7	Mr.X (Dec 6 2021)	If only one or two people have returned after the radiation decontamination work completion, anyone will judge that residents do not hope to return. People would say that there was no need to go to the trouble of spending taxpayers' money to decontaminate the district. I am afraid to be blamed by the government or the public for why Nagadoro residents are being selfish and not returning to their homes despite the progress/completion of radiation decontamination work.	People would say that there was no need to go to the trouble of spending taxpayers' money to decontaminate the district; why Nagadoro residents are being selfish and not returning to their homes despite the progress/completion of radiation decontamination work.	unfruitful radiation decontamination work	the contraposition of ordinary and Nagadoro people; unfruitful radiation decontamination work for ordinary people; non-return; criticizing the Nagadoro residents	decontamination as a waste of taxpayers' money; doubt among people in and outside of Iitate village toward Nagadoro residents	
8	Mr.X (Dec 6 2021)	The trouble is that the decontamination is done for residential areas, not farmland. Residents like us who have farmland outside residential areas will not be able to use their farmland even if the decontamination is completed. This is because the farmland is still contaminated.	Residents like us who have farmland outside residential areas will not be able to use their farmland even if the decontamination is completed.	decontamination performed only to a limited extent	those with/without farmland in their residential area; decontamination only of a residential area; farmland that cannot be used; limited decontamination; farming which cannot be resumed; Specified Reconstruction and Revitalization Zone	radiation decontamination only in and around the specified reconstruction and revitalization base; Nagadoro including suitable and safe, and dangerous places for food production; difficulties in earning a livelihood	
9	Mr.X (Nov 11 2021)	Once the evacuation order is lifted, many subsidies or support programs will no longer be available. Why? Because the lifting means the completion of radiation decontamination work and thus that the district became a "returnable" place for residents. Residents must live by their own effort even though many places in the Nagadoro district are considered unsuitable for living and farming due to the absence of decontamination work. The government has abandoned its support for the residents.	many subsidies or support programs will no longer be available;unsuitable for living and farming; The government has abandoned its support for the residents.	completion of decontamination and end of evacuation as inseparable prerequisites; irresponsible country	lifting the evacuation order because decontamination is complete; loss of status as evacuees; termination of government assistance; government policy of abandonment	lift of evacuation order with assumption of completion of decontamination; loss of status as evacuees; end of government's assistance residents' received; government's abandonment policy	
10	Mr.X (Nov 11 2021)	Suppose that rice cultivation is resumed in the Nagadoro district. Residents in other districts of Iitate would say, "Don't indicate rice grown in Nagadoro as a product of Iitate. If rice from Nagadoro is distributed, customers may feel uncomfortable, and thus sales of Iitate rice will be damaged." If they say so, we cannot continue farming.	"Don't indicate rice grown in Nagadoro as a product of Iitate. If rice from Nagadoro is distributed, customers may feel uncomfortable, and thus sales of Iitate rice will be damaged."	regional radiation dose gaps between districts within Iitate Village; discriminatory treatment of the Nagadoro products	discrimination in the local community; reputational damage; divisions within the village	discriminatory treatment of products/residents; conflicts and divisions within the villagers due to resumed farming	
11	Mr.X (Nov 11 2021)	Everyone is really getting older. Then, the generation that supports the community and agriculture, younger people than me, are hardly found. For example, there are only two people in their 30s.	Everyone is really getting older; younger people than me, are hardly found.	Nagadoro district with many elderly people; few younger generations who bear the future	low birthrate and ageing population; depopulation and marginalized communities; small working population	community which has many older adults and few young people; shortage of agricultural workers	
12	Mr.X (Nov 11 2021)	Each household did not educate their children and young people about agriculture. For example, we have not taught them the skills and knowledge necessary to cut weeds in the rice paddies with a back-powered mower or work with a tractor.	Each household did not educate their children and young people about agriculture.	failure to train successors; lack of experienced farmers; concerns about future land management	generativity and education; future time perspective	failure or lack of successor training	

Storyline	
Theory writing	
Further investigation	

SCAT(Steps for Coding and Theorization)

Downloaded form from SCAT WEB site scatform-eng.xls
http://www.educa.nagoya-u.ac.jp/~otani/scat/scatform-eng.xls

Note:
Of the 30 texts data included in the analysis, four examples were extracted by each interview schedule.
For readability, items "<5>questions & tasks", "Storyline", "Theory writing", and "Further investigation" were omitted; these items were either mentioned in the manuscript body or were redundant as a description of the coding procedure.
Data were ordered by the interview schedule (latest) and appearance within each interview.
In SCAT, although storylines should be described using the terms in item <4>, such terms revised and then used in storylines of the manuscript due to the grammatical requirements if necessary.
