# Supplementary material for: Concerns related to returning home to a “difficult-to-return zone” after a long-term evacuation due to Fukushima Nuclear Power Plant Accident: A qualitative study
Source: PLoS One. 2022 Aug 29;17(8):e0273684. doi: 10.1371/journal.pone.0273684 (PMC9423635; doi:10.1371/journal.pone.0273684)
Supplement: S2 Table — (PDF) [file pone.0273684.s002.pdf]

Supplement S2 Table. The components of storylines

<4> themes, constructs in considerations of context	Content (storyline) #
impossibility of rebuilding and maintaining the community	4
the passage of time since the FDNPP accident	4
long-term prohibition of entry due to its designation as a difficult-to-return zone	4
significant deterioration and collapse of their former dwellings	4
the cost of rebuilding new dwellings to live in after their return	4
the rational decision of returning or not	4
Nagadoro district as an agriculture-oriented area	1
prospects that residents hardly return	1
wells and river water at risk of radiation contamination	2
the difficulty of securing water sources	2
unsustainable livelihoods	2
the unfamiliarity with information technology among older adults	1
group farming by families and other groups	1
unsustainable agriculture	2
poor management and discord regarding a cooperative farming system in the past	1
the lack of successful experience in a cooperative farming system	1
Nagadoro district as a model case or touchstone for promoting a return	3
the obstacle to decontamination and reconstruction efforts in other evacuation zones	3
decontamination work as a government project funded by taxes	3
evaluation by the number of people who return	3
decontamination as a waste of taxpayers' money	3
doubt among people in and outside of Iitate village toward Nagadoro residents	3
radiation decontamination only in and around the specified reconstruction and revitalization bas	4
Nagadoro including suitable and dangerous places for food production	2
difficulties in earning a livelihood	1
completion of decontamination as a fait accompli	3
practical forcing to return home	4
Nagadoro residents treated as villains or scapegoats	3
lift of evacuation order with an assumption of completion of decontamination	4
loss of status as evacuees	4
end of government's assistance residents' received	4
government's abandonment policy	4
high radiation levels near homes, farmland, and other places	4
discriminatory treatment of products/residents	2
Nagadoro residents as eccentrics	2
conflicts and divisions within the villagers due to resumed farming	2
a community which has many older adults and few young people	1
shortage of agricultural workers	1
failure or lack of successor training	1

Note:
The storyline was constructed based on 39 themes (codes) extracted by abstracting the narrative.
The number of texts is inconsistent with the number of themes since multiple themes could be generated from a single text.
In SCAT, although storylines should be described using the terms in item <4>, such terms revised and then used in storylines of the manuscript due to the grammatical requirements if necessary.
